# Supplementary material for: Holographic intravital microscopy for 2-D and 3-D imaging intact circulating blood cells in microcapillaries of live mice
Source: Sci Rep. 2016 Sep 8;6:33084. doi: 10.1038/srep33084 (PMC5015119; doi:10.1038/srep33084)
Supplement: Supplementary Information [file srep33084-s9.pdf]

Holographic intravital microscopy for 2-D and 3-D imaging intact circulating blood cells in microcapillaries of live mice

Kyoohyun Kim[1,2+], Kibaek Choe[2,3+], Inwon Park[2,4], Pilhan Kim[2,3*] and YongKeun Park[1,2,5,*]

[1]Department of Physics, Korea Advanced Institute of Science and Technology (KAIST), Daejeon 34141, Republic of Korea

[2]KI for Health Science and Technology (KIHST), KAIST, Daejeon 34141, Republic of Korea

[3]Graduate School of Nanoscience and Technology, KAIST, Daejeon 34141, Republic of Korea

[4]Graduate School of Medical Science and Engineering, KAIST, Daejeon 34141, Republic of Korea

[5]Tomocube, Inc., Daejeon 34051, Republic of Korea
[+]These authors contribute equally to this work.

*Correspondence:
Prof. YongKeun Park (yk.park@kaist.ac.kr), Prof. Pilhan Kim (pilhan.kim@kaist.ac.kr)

SUPPLEMENTAL MATERIAL

Supplementary Movie 1
Time-lapse images of raw holograms, optical phase maps before and after applying the scattering reduction method (lower row), bright field, phase contrast, and differential interference contrast microscopic images (upper row). The bright field, phase contrast, and differential interference contrast microscopic images are emulated using the measured holograms.

Supplementary Movie 2
Two-dimensional time-lapse intravital quantitative phase images of individual red blood cells (RBCs) flowing through the microvasculature of the live mouse mesentery in the field of view of 26.0 μm × 26.0 μm. Dry mass of individual RBCs is measured from each phase image, and colorbar indicates dry mass densities (pg/μm^2) of haemoglobin inside RBCs.

Supplementary Movie 3
Two-dimensional time-lapse intravital quantitative phase images of the bloodstream flowing through the microvasculature of the live mouse mesentery. Total dry mass of RBCs in the field of view of 29.2 μm × 29.2 μm is measured from each phase image, and colorbar indicates dry mass densities (pg/μm^2) of haemoglobin inside RBCs.

Supplementary Movie 4
Two-dimensional time-lapse intravital quantitative phase images of the bloodstream flowing through the bifurcating microcapillaries of the live mouse mesentery in the field of view of 51.6 μm × 57.2 μm. Colorbar indicates dry mass densities (pg/μm^2) of haemoglobin inside red blood cells.

Supplementary Movie 5
Two-dimensional time-lapse intravital quantitative phase images of individual RBCs flowing through the microvasculature of the live mouse mesentery in the response to the injection of phosphate-buffered saline solution. Time-lapse quantitative phase images are measured for an hour for every 15 minutes. The field of view is 57.2 μm × 57.2 μm.

Supplementary Movie 6
Two-dimensional time-lapse intravital quantitative phase images of individual RBCs flowing through the microvasculature of the live mouse mesentery in the response to the injection of lipopolysaccharide solution. Time-lapse quantitative phase images are measured for an hour for every 15 minutes. The field of view is 57.2 μm × 57.2 μm.

Supplementary Movie 7
The *x*-*y* cross-sectional slices of the time-lapse three-dimensional (3-D) refractive index (RI) distribution of individual RBCs flowing through the microcapillary in the field of view of 30.6 μm × 32.9 μm. Colorbar indicates the RI values of RBCs.

Supplementary Movie 8
Rendered isosurfaces of the time-lapse 3-D RI distributions of RBCs flowing through the microcapillary in the field of view of 30.6 μm × 32.9 μm.

Supplementary Movie 9
Rendered isosurface of the microcapillary with various viewing angles.
